# Next generation data systems and knowledge products to support agricultural producers and science-based policy decision making

**DOI:** 10.1016/j.agsy.2016.10.009

**Published:** 2017-07

**Authors:** Susan M. Capalbo, John M. Antle, Clark Seavert

**Affiliations:** Department of Applied Economics, Oregon State University, United States

**Keywords:** Data systems, Knowledge products, AgBizLogic, TOA-MD, Next generation

## Abstract

Research on next generation agricultural systems models shows that the most important current limitation is data, both for on-farm decision support and for research investment and policy decision making. One of the greatest data challenges is to obtain reliable data on farm management decision making, both for current conditions and under scenarios of changed bio-physical and socio-economic conditions. This paper presents a framework for the use of farm-level and landscape-scale models and data to provide analysis that could be used in NextGen knowledge products, such as mobile applications or personal computer data analysis and visualization software. We describe two analytical tools - AgBiz Logic and TOA-MD - that demonstrate the current capability of farmlevel and landscape-scale models. The use of these tools is explored with a case study of an oilseed crop, *Camelina sativa*, which could be used to produce jet aviation fuel. We conclude with a discussion of innovations needed to facilitate the use of farm and policy-level models to generate data and analysis for improved knowledge products.

## Highlights

• Better data are needed to support the development of improved knowledge products for policy decision making.
• Farm-level decision models can be used to support farm decision making and to acquire data for landscape-scale policy analysis.
• A farm-level model (*Ag Biz Logic*) and an economic impact assessment model (TOA-MD) illustrate possible model linkages.
• A case study of biofuel adoption illustrates how these models could be used to improve data acquisition and analysis linked to knowledge products.

## 1 Introduction

In the introduction to this special issue, Antle et al. (2016b) discuss the critical need for data, models and knowledge products that provide user-friendly data acquisition and analytical capability for decision makers. The use cases range from farm-level decision support, to the agricultural research community and donors making research investment decisions, to policy decision makers whose goal is the sustainable management of natural resources. Janssen et al. (2017) provide examples of data and information technology structures that illustrate how private and public data components could be developed for such use cases. Jones et al. (2017) argue that the most important current limitation is data, both for on-farm decision support and for research investment and policy decision making. One of the greatest data challenges is to obtain reliable data on farm management decision making both for current conditions and under scenarios of changing bio-physical and socio-economic conditions.

This paper discusses how farm-level decision models can be used to support farm decision making and to provide data for landscape-scale models for policy analysis. In the second section of this paper we provide an overview of the kinds of information needed to support science-based policies for sustainable landscape management as well as improved on-farm management. We describe how existing decision support tools could be used to develop a data infrastructure that can provide this type of information. In sections three and four we describe a landscape-scale policy analysis tool (TOA-MD) and a farm-level decision support tool (AgBiz Logic) that could be used to support landscape scale and farm level decision-making. Section five illustrates the use of these tools with an analysis of the economic potential for a new oilseed crop, *Camelina sativa*, to be incorporated into the winter wheat-fallow system used in the U.S. Pacific Northwest. In the concluding section we discuss the challenges that will need to be addressed if these and other similar data and modeling tools are to be integrated into data and modeling platforms that could support new knowledge products for both farm and policy decision makers.

## 2 The need for better data, models and knowledge products

Both governmental and non-governmental organizations have established a wide variety of data, knowledge and institutional arrangements that together constitute an “infrastructure” that supports management of agricultural landscapes. This physical and institutional infrastructure differs greatly around the world, but all have in common the very substantial challenge of acquiring timely, site-specific data and combining it with analytical tools to improve the quality of decision making from farm to landscape scales. To varying degrees, this decision making infrastructure has evolved in many countries along with public policy towards what we will describe as “science-based policy” – that is, policy designed to achieve the goal of sustainably managing agricultural landscapes as efficiently and effectively as possible given the best-available science and technology.

A large and growing body of scientific knowledge from agricultural, environmental, economic and social science disciplines exists as a foundation on which a science-based policy for agriculture can be further advanced, starting with the idea that agriculture is a “managed ecosystem” (Antle et al., 2001, Antle and Capalbo, 2002, Swinton et al., 2007). The scientific literature has established that farmers' land management decisions affect biological and physical systems through a number of mechanisms. Some effects, such as changes in soil productivity, may be limited to the land owned by the farmer; others, such as runoff into surface waters, may appear offsite. A key insight from this body of scientific literature is that agricultural productivity depends upon and plays a key role in providing a set of “ecosystem services” ranging from food production to the provision of clean water and maintenance of biodiversity (Reid et al., 2005).

There are two types of policies and programs being used for agricultural landscape management often referred to as “conservation” and “working lands” policies, closely related to the ideas of “land sparing” and “land sharing” used by ecologists for wildlife management (Phalan et al., 2011). In addition to managing agricultural landscapes, agricultural policy in many countries has also sought to improve the economic well-being of agricultural households through a variety of subsidy programs that transfer income from taxpayers to agricultural producers and landowners. The biofuel policy we discuss later in this paper is an example of a working land policy designed to produce environmental benefits by substituting biofuels for fossil fuels while maintaining food crop production.

These and other types of domestic and trade policies may affect producers' land management decisions, and may either complement or conflict with the goals of sustainably managing agricultural landscapes. For example, the biofuel development program investigated later in this paper shows that subsidies may be required to achieve its goals of increasing biofuel crop production, but may also reduce food crop production and increase food prices. Both the resource efficiency and the distributional effects of policies are important to agricultural producers and to others in society, and need to be taken into account in designing science-based policies. Indeed, there are inevitably trade-offs among the various private and public goals related to the management of agricultural landscapes. A goal of the knowledge infrastructure needed to support science-based policy is to improve our understanding of these trade-offs so that stakeholders can make informed choices among policy alternatives and their likely impacts.

### 2.1 Assessing policy synergies and tradeoffs

Economics provides an analytical framework to evaluate the need for policy interventions, given sufficient physical, biological and economic data. In this framework, typically described as “benefit-cost analysis,” private outcomes (e.g., farm income generated by producing and selling crops and livestock) are combined with the value of “non-market” outcomes, such as maintaining water quality and biodiversity, to determine the management strategy that yields the best outcome for society. In principle, if all policy options could be evaluated in this way, the best option could be identified. To implement this benefit-cost framework, however, both quantities and values of marketed goods are needed (e.g., quantity and price of corn produced), as well as quantities and values of non-market outputs (e.g., nutrient concentration in surface water and the environmental or health damages caused by it).

While it is straightforward to measure and value market outcomes such as the amount and value of corn produced in a given area, it is difficult to quantify and value non-market outcomes such as changes in ecosystem. With adequate scientific understanding, spatially-relevant data and suitable measurement technologies, it is possible to objectively quantify the non-market. But in many cases valuing non-market outputs is exceedingly difficult. For example, contamination of water by nutrients such as nitrates may have adverse impacts on human health, and it may be possible to estimate the magnitude of these effects, but it is difficult to attach a monetary value to health effects that is generally accepted by the affected people and society. Similarly, ecosystem services such as biodiversity are difficult to quantify and value in monetary terms. For these reasons, strict application of the “benefit-cost analysis” approach to the design of science-based policies faces serious challenges.

An alternative to benefit-cost analysis is what we refer to as “policy tradeoff analysis” (Crissman et al., 1998, Antle et al., 2014, Kanter et al., 2016). Rather than attempting to attach monetary values to ecosystem services, the tradeoff analysis approach defines a set of quantifiable economic, environmental and social “indicators” that can be used to assess the status of the agricultural landscape and outcomes associated with it. Alternative policies are evaluated in terms of the interactions among these indicators. In this approach, there is no one “solution” or best policy because different stakeholders may value tradeoffs between outcomes (indicators) differently. However, the tradeoff analysis approach has the virtue of providing the various stakeholders with the information they need to make these value judgments.

Tools suitable for policy tradeoff analysis are already being used in research and policy design (Antle et al., 2014, Kanter et al., 2016). Many indicators have been developed for policy analysis (Bates and Scarlett, 2013). Various measures of farm household well-being are used, such as farm income and its distribution among geographic regions and among different types of farms. Measures of environmental outcomes and ecosystem services are available from direct measurements and from models, including soil quality and productivity, air and water quantity and quality, greenhouse gas emissions, and wildlife habitat. For example, the U.S. Department of Agriculture has constructed an “environmental benefits index” to assist in the design and implementation of conservation programs that combines a number of different environmental indicators into a summary measure (U.S. Department of Agriculture. Economic Research Service, 2006).

### 2.2 The need for better farm-level data and analytical tools

The increasing utilization of precision farming and mobile technologies, together with improvements in data management software, offer expanding opportunities for an integrated data infrastructure that links farm-level management decisions to site-specific bio-physical data and analytical tools to improve on-farm management. These farm-level data can be integrated with public data at the landscape scale for research and policy analysis. Analytical tools using data at the landscape scale could provide the improved understanding needed to support science-based policy and sustainable management of agricultural landscapes.

Much of this growing volume of new data is private – for example, information about where and when agricultural operations occur, and their consequences. There is also a growing amount of public data, such as satellite imagery and weather and soil data, historical crop yields, and economic data. A critical feature of the new knowledge infrastructure is that it must be able to measure, store, manage and integrate both private and public data in ways that respect the privacy and proprietary interests of individuals while enabling diverse stakeholders to benefit from improved information and analyses.

In addition to the need to be profitable and provide an acceptable standard of living for the farm household, farm decision making must increasingly respond to the requirements of environmental regulations and related public policies aiming to achieve more sustainable resource management. Farmers must also meet the demands by food companies and the public for assurance that sustainable and ethical practices are being used. All of these pressures – economic, environmental and social – create a need for better farm-level data and analytical tools.

New technologies began to provide new sources of “big data” for farm management beginning with the automation of agriculture 1990s. Machinery including tractors, chemical applicators, and harvesters are now equipped with global positioning system controllers that can both control and track various aspects of the farm operations, and hand-held mobile devices as well as personal computers and management software provide managers with ways to enter information about management decisions and carry out analysis. Moreover, these data can be stored “in the cloud,” aggregated with data from many operations, and used for analysis to improve on-farm management as well as for policy analysis as discussed in the previous section.

Due to these technologies, some producers now have access to their past crop yield and related management data at the field or sub-field resolution. This information can be combined with satellite imagery, high-resolution spectral and thermal data obtained from UAVs, and weather data. These data provide the foundation for highly sophisticated, site-specific management – i.e., “precision agriculture” – that has the potential to substantially improve economic and environmental efficiency of management decisions and also provide the kind of information needed to meet both private and public demands for sustainable agricultural production. However, to achieve these efficiency improvements, the capability to effectively capture and analyze these data is needed. For example, despite these advances in data acquisition by equipment sensors, variable rate application of nutrients and other agricultural chemicals continues to be based on simple rule-of-thumb or empirical approaches, and not by using model-based systems approaches that account for the interaction of soils, weather and related management decisions. In addition to the farm and landscape scale analyses discussed thus far, there will also be a growing demand for farm-level information to be integrated with other components of the agricultural value chain, to meet both policy requirements and consumer demands for more sustainably produced food products.

### 2.3 Assessing impacts of new technologies and climate adaptation

Most agricultural technology impact assessment is carried out after technologies have been disseminated. However, there is a growing recognition of the need for forward-looking, or *ex ante*, technology impact assessment designed to anticipate both intended and unintended impacts (Antle, 2011, Antle et al., 2014). One of the most important growing applications of *ex ante* impact assessment is for climate adaptation and climate smart agriculture (Lipper et al., 2014). There is a widely recognized need to not only assess climate impacts on agricultural systems, but also to develop adaptation strategies and provide information to support farmer decision making for climate adaptation (Government Accounting Office, 2014). There are two key elements of this type of analysis.

First, the research team must project the future climate and socio-economic conditions in which the farm decision maker will be operating. New multi-disciplinary and participatory methods to create future scenarios for this type of analysis have recently been developed (Valdivia et al., 2015). Second, researchers need to obtain information about the potential adaptations and the ways that farm decision makers would implement them. Farm-level decision support tools linked to a web-based system could be used to obtain reliable information about famers' current management practices, and also could be used to obtain their evaluations of management alternatives under conditions defined by future changes in climate, economic conditions and policies.

### 2.4 Farm-level and landscape-scale data and analytical tools

**Fig. 1:**
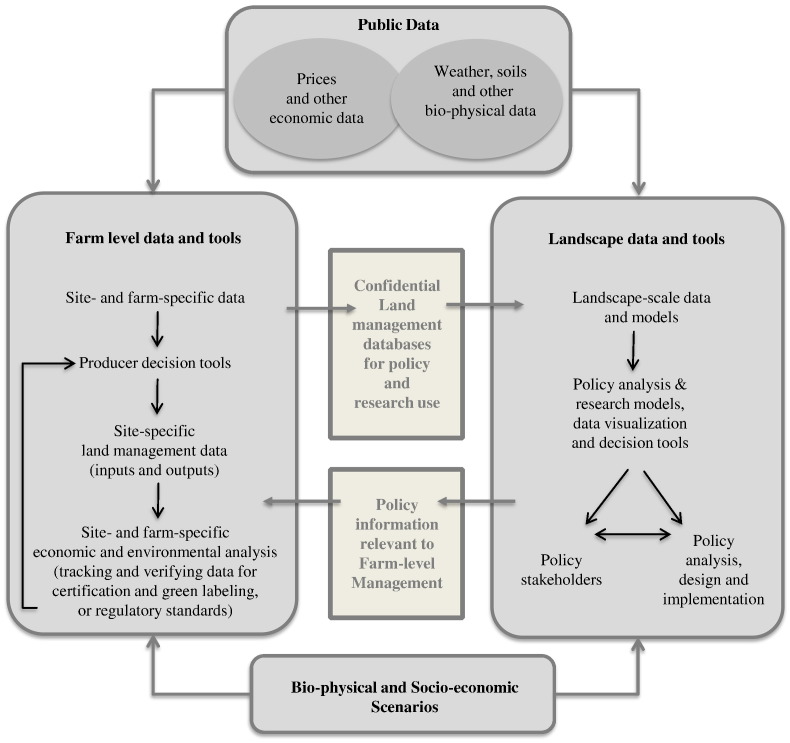
Linkages between Data and Decision Tools at Farm and Landscape Scales (source: Antle et al., 2015).

Fig. 1 provides an overview of the features of farm-level data and decision tools, landscape-scale data and analytical tools that support science-based policy, and their interrelationships. While farm-level decision making and landscape-scale analysis have different purposes, they both depend on both private data (site- and farm-specific characteristics of the land and the farm operation, and the site- and farm-specific management decisions that are made) as well as public data (weather, climate and other physical data describing a specific location, and prices and other publicly available economic data). A key question for the design of the agricultural knowledge infrastructure is how both types of data can be collected, managed and utilized efficiently and securely.

Farm-level data and decision tools are evolving rapidly along with innovations in computer power, software, mobile information technologies and technologies for site-specific management. The left-hand side of Fig. 1 presents the generic structure of these tools, the data they use as inputs, and the outputs that are generated. Various decision tools and software are now in use (Antle et al., 2015b) which collect detailed information and generate outcomes that are useful for farm-level management decisions. This information and data can be used to monitor the economic and environmental performance of a farm operation over time and space.

The right hand side of Fig. 1 shows the general structure of the data and models needed to carry out landscape-scale research and policy tradeoff analysis. There are three broad categories of regional data: publicly available biophysical data, including down-scaled climate and soils data; publicly available economic data, including prices and policy information; and the confidential site- and farm-specific data obtained from producer- and industry-generated databases.

As with farm-level decision tools, there is a need to more systematically develop and apply methods for the improvement of these models, for example through model inter-comparison studies such as those being undertaken by the Agricultural Model Inter-comparison and Improvement Project. Typically these models require spatially and temporally explicit data that are statistically representative of the farms and landscapes in a geographic region in order to provide reliable information about economic and environmental impacts and tradeoffs.

The currently available data are inadequate for various reasons. Many model implementations rely on the publicly available information on land management collected periodically through mailed questionnaires or enumerator interviews, which usually limits the spatial dimension of the models to political units, agro-ecological zones or similar delineations. Consequently, models often must be operated with averaged data that may fail to accurately represent site-specific environmental processes and outcomes. Many data are collected with samples that are not statistically representative of relevant regions or populations for landscape-scale analysis; many data are not spatially or temporally explicit, are only available (released) after substantial aggregation (thus limiting their usefulness), and are often available with long time lags between when the land management decisions are made, the data are collected, and when they become available for research or policy purposes. For example, the 2012 U.S. agricultural census data were only available in 2014, and then are only available in limited ways for research and policy analysis. Longitudinal data are particularly important for policy research, i.e., representative samples of farms that provide data for the same farms over time. The Living Standards Measurement Survey data being coordinated by the World Bank are being collected longitudinally in some countries now, but due to issues such as long respondent recall and limited statistical representation, these data have a number of substantial limitations. Another critical issue is data quality. Farmers lack incentives to bear the high costs of responding to lengthy questionnaires, and often lack detailed records needed to accurately respond to detailed questions about management inputs, production outputs, and prices paid and received, and various other details often asked in farm surveys. A tool that could be used by farmers to make management decisions, and simultaneously collect that information for research and policy analysis, could overcome these limitations.

## 3 TOA-MD: a model for landscape-scale data integration and policy tradeoff analysis

Landscape-scale policy analysis can be implemented using various spatially-explicit models designed to simulate the adoption and impact of new technologies, changes in policy, and environmental change such as climate change (Antle and Capalbo, 2001, Troost and Berger, 2014, van Wijk et al., 2014; and Kanter et al., in press). In this section we briefly describe an economic impact assessment model called TOA-MD (Tradeoff Analysis Model for Multi-dimensional Impact Assessment). TOA-MD provides a framework in which bio-physical and economic data can be integrated for technology impact assessment and policy analysis at the landscape (or population) scale.

**Fig. 2:**
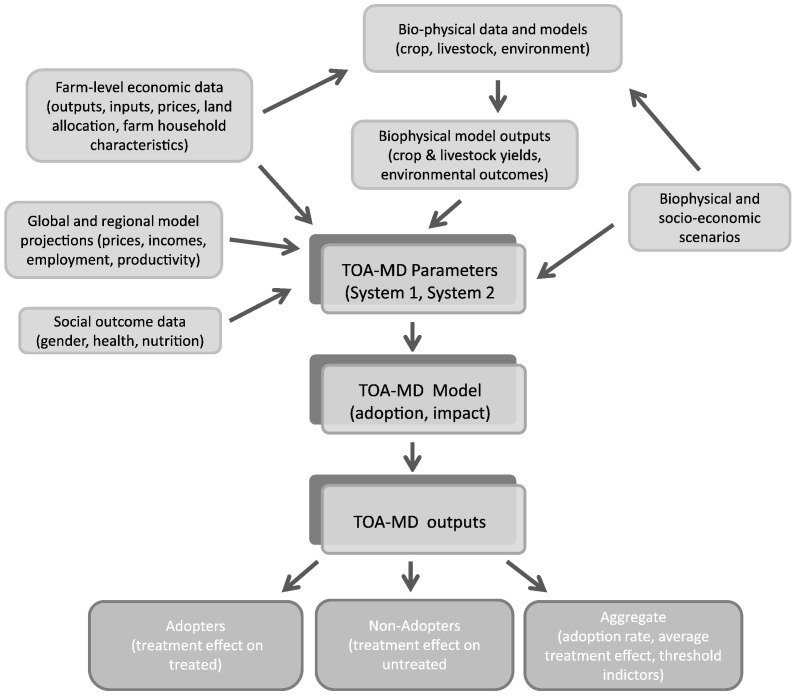
TOA-MD model data inputs and outputs.

The TOA-MD model is a parsimonious, generic model for analysis of technology adoption and impact assessment, and ecosystem services analysis. Further details on the conceptual and statistical foundations of the model are provided in Antle (2011) and Antle et al. (2014). The model software and the data used in various studies are available to researchers with documentation and self-guided learning modules at http://tradeoffs.oregonstate.edu. Various types of data can be used to implement an analysis using TOA-MD and other landscape-scale policy analysis models. The data can include farm production data, simulated outputs of bio-physical models, price projections from global or national market models, and data from alternative policy or climate scenarios, depending on the type of analysis (Fig. 2). Estimation of parameters for TOA-MD and other spatially-explicit impact assessment models requires data from a statistically representative sample of the farm population, as discussed in Antle and Capalbo (2001) for econometric models, and in Troost and Berger (2014) for models based on mathematical programming.

The TOA-MD model was designed to simulate technology adoption and impact in a population of heterogeneous farms. The TOA-MD model uses the standard economic model that is the foundation of the econometric policy evaluation literature (Heckman and Vytlacil, 2007). The analysis is applied to farm decision makers who choose between the production system they are currently using (call this System 1) and an alternative production (System 2). Each decision maker is assumed to choose the system with the highest expected return. Thus, in the population, the proportion of farmers using system 2 is determined by the distribution of the difference in expected economic returns between the two systems. Other impacts (environmental or social) are estimated based on the statistical relationship between those variables and expected economic returns to the alternative systems. The outputs of the TOA-MD model include the predicted adoption rate of the alternative system, the average impacts on adopters, and the average impacts on the entire population of farm households. The model can also generate indicators showing the percent of households experiencing an outcome above or below a defined threshold. An example of a threshold indicator is a poverty rate showing the percent of households with incomes below a poverty line.

The analysis of technology adoption and its impacts depends critically on how the effects of the new technology interact with bio-physical and economic conditions faced by farm decision makers. A key element in the TOA-MD analysis is reliable estimates of the effect of the new technology (i.e., the changes in the farming system that farmers could adopt) on the farming system's productivity and profitability. This information can come from various sources, including from formal crop and livestock simulation models, from experimental or observational data, or from expert judgment.

Two types of tradeoff analysis that can be carried out with TOA-MD are described by Antle et al. (2014) as adoption-based tradeoffs and price-based tradeoffs. Adoption-based tradeoffs occur when the adoption rate of a technology changes in response to an economic incentive or other factor affecting technology adoption. An important example of an adoption-based tradeoff is a policy to provide payments to farmers if they change management practices to increase the provision of ecosystem services such as soil carbon sequestration. Price-based tradeoffs occur when changes in the prices of the outputs or inputs used by farmers induce them to make changes in their land management decisions that in turn induce changes in the economic, environmental or social outcomes associated with the farming system. The analysis of *Camelina sativa* presented below is an example of a price-based tradeoff.

## 4 *AgBiz Logic*: a farm-level data acquisition and analysis tool

**Fig. 3:**
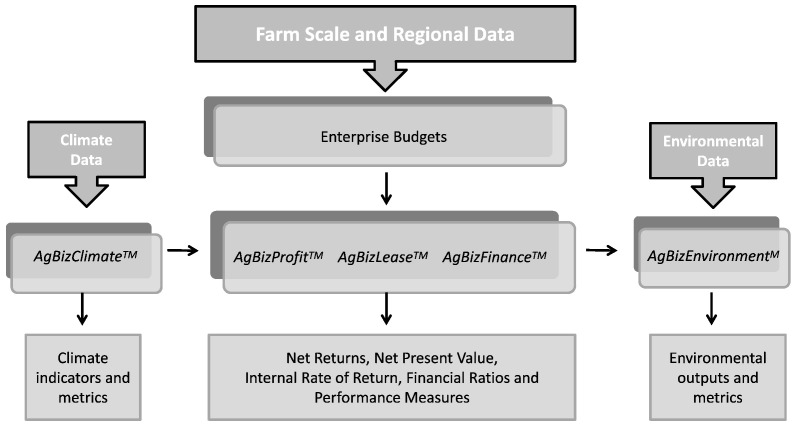
*AgBiz Logic* data inputs, model components and outputs.

*AgBiz Logic* (AgBiz Logic.com) is an analytical tool that integrates data, scenarios, economic and financial calculators and climate and environmental modules. It generates estimates of economic and environmental outcomes for current and alternative management practices (Fig. 3). A key feature of *AgBiz Logic* that distinguishes it from many other farm management tools is that it is designed to analyze current and prospective management scenarios. This feature makes it a potentially useful tool to acquire data for a landscape-scale analytical tool such as TOA-MD.

### 4.1 Components of *AgBizLogic*

The *AgBiz Logic* software suite consists of the following economic and financial modules:

• *AgBizProfit:* capital investment tool that evaluates an array of short-, medium-, and long-term investments. The module uses the economic concepts of net present value, annual equivalence, and internal rate of return to analyze the potential profitability of a given investment.
• *AgBizLease:* a module to establish alternative short- and long-run crop, livestock and other capital investment leases. The module uses the economic concepts of net present value to analyze crop sharing or rental agreements under these alternatives.
• *AgBizFinance:* a module for making investment decisions based on financial liquidity, solvency, profitability, and efficiency of the farm or ranch business. After an *AgBizFinance* analysis has been created, investments in technology, conservation practices, value-added processes, or changes to cropping systems or livestock enterprises can be added to or deleted from the current farm and ranch operation. Changes in financial ratios and performance measures are also calculated.
• *AgBizClimate:* a module that translates information about climate change to farmers and land managers that can be incorporated into projections about future net returns. By using data unique to their specific farming operations and locations, growers can design management pathways that best fit their operations and increase net returns under alternative climate scenarios.
• *AgBizEnvironment:* a module that uses environmental models and other ecological accounting to quantify changes in environmental outcomes such as erosion, soil loss, soil carbon sequestration and GHG emissions associated with input levels and management practices.

*AgBiz Logic* operates on the premise that growers want to maximize net returns over time, taking into account investment costs, operating expenses and revenues for crop and livestock products. This decision support tool has been used to quantify farm-scale tradeoffs associated with changes in climatic conditions. Capalbo et al. 2017 present an illustrative analysis of how climate change may impact dry-land wheat producing farmers in the U.S. Pacific Northwest. Projected changes in climate are translated into changes in key climate factors affecting the grower's yields via the *AgBizClimate;* these yield changes are transformed into net returns. These yield changes are the impetus for producer-generated adjustments in input use, management, and technology adoption. Decision tools and modules such as *AgBiz Logic*; provide essential analytical output for efforts labeled climate-smart agriculture which focuses on making farms and farmers more resilient to a changing climate. These decision support tools are at the very heart of the recommendations called for in the recent U.S. Government Accountability Office report 14–755 (U.S. GAO, 2014), which speaks to USDA's ongoing efforts to better communicate information to growers in a timely downscaled manner.

### 4.2 AgBizLogic as a data acquisition tool

One of the greatest challenges in implementing policy analysis of alternative agricultural systems, such as adaptation to climate change, or responses to new policies or technologies, is the design of plausible “counter-factual” (i.e., as-yet unobserved) systems. *AgBiz Logic* is designed to be a farm-level scenario analysis tool, where the scenarios can involve any type of alternative management. This feature makes *AgBiz Logic* uniquely suited to serve as a data generation tool for policy analysis using a model like TOA-MD that requires data for current as well as prospective or future systems. *AgBiz Logic* provides a systematic framework in which farm decision makers can record their best estimates of the cost and productivity effects of a new system on their own farms. If this information could be acquired from a suitable sample of farms, it could then be used by analysts to estimate parameters of the TOA-MD model for landscape-scale policy analysis.

The conventional way to obtain the farm production data is to conduct a survey, such as the surveys done periodically by government agencies (e.g., agricultural census or other statistical surveys such as the Agricultural Resource Management Survey in the United States or the Farm Accountancy Data Network data collected in European Community countries). There are various limitations to farm survey data. One is that the data are often collected periodically, e.g., the U.S. agricultural census is carried out on five-year intervals, and then only made available to researchers with a substantial delay. Another major limitation is that the data often lack sufficient detail, particularly for management decisions such as fertilizer and chemical use, machinery use, and agricultural labor. A third limitation is that these surveys can be extremely expensive both for respondents (e.g., to complete large elaborate questionnaires) and for organizations collecting the data (e.g., to employ enumerators, data entry workers, quality control specialists, etc.).

A tool like *AgBiz Logic* could be utilized to provide higher quality, more timely data at lower cost. As portrayed in Fig. 1, a data system that linked farm management software to a confidential database could provide near real-time data on management decisions, and do so for a statistically representative “panel” of farm decision makers over time. Moreover, the level of detailed management data utilized by *AgBiz Logic* would provide the needed level of detail for implementation of analysis using a tool such as TOA-MD. Also, users of *AgBiz Logic* would have every incentive to enter accurate information because they would be using this information to make their actual management decisions. Finally, a tool like *AgBiz Logic* provides a user-friendly, efficient way for farmers to enter data, thus substantially reducing the cost of data collection.

## 5 Potential of *Camelina sativa* as a biofuel in the U.S. Pacific Northwest

In this section we illustrate the use of TOA-MD to evaluate *Camelina sativa* for its potential use as a crop that could produce biodiesel fuel for aviation and other uses, particularly in regions where dryland cropping systems are currently dominant. Our goal is to illustrate the type of analysis that could be implemented using data that could be generated by a tool like *AgBiz Logic.*

The policy question addressed in this example is whether it would be economically feasible to incorporate *Camelina* into the dryland wheat system currently in use in the U.S. Pacific Northwest (PNW) as part of the U.S. Department of Energy's “Farm to Fly” initiative. Key issues for this initiative are the profitability of *Camelina* for farmers at prices competitive with fossil fuels, whether it would be possible to provide sufficient quantities to meet the goals of the private airline industry and the U.S. military, and what impacts biofuels would have on food production and prices.

Table 1 summarizes the farm level revenue and cost data used in this example. These data were obtained from farmers' responses to the 2007 Agricultural Census, but similar data could have been obtained using *AgBiz Logic* from a statistically representative sample of farms. We implement the analysis using agricultural census data to illustrate the analysis that could be done using similar data obtained from *AgBiz Logic*.

**Table 1:** Revenue and cost statistics for analysis of *Camelina* adoption based on 2007 agricultural census data for winter wheat-fallow system and representative budget data and yield experimental data for *Camelina.*

Farm size		Wheat yield (bu/acre)	Wheat revenue ($/farm)	Other crops revenue ($/farm)	Wheat and other crops cost ($/farm)	Govt. subsidies ($/farm)	*Camelina* yield (lbs/acre)	*Camelina* revenue ($/farm)	*Camelina* cost ($/farm)
System 1 (Winter wheat - fallow rotation)									
Large	Mean	50	473,095	15,341	273,879	60,744			
	Std Dev	15	206,054	15,427	199,556	33,640			
Small	Mean	51	65,360	4921	69,219	12,827			
	Std Dev	18	42,245	10,626	55,755	8887			

System 2 (Winter wheat - *Camelina* rotation)									
Large	Mean	32	307,512	15,341	273,879	60,744	1400	532,000	284,050
	Std Dev	50	133,935	15,427	199,556	33,640	n.a.	231,710	123,716
Small	Mean	33	65,360	4921	69,219	12,827	1400	98,000	87,500
	Std Dev	12	27,459	10,626	55,755	8887	n.a.	41,172	36,761

Note: mean large farm size = 4170 acres, mean small farm size 720 acres.

Wheat is produced in the PNW in various rotations with fallow and with other crops. This analysis involves incorporating *Camelina* into the winter wheat-fallow (WWF) system practices in low-rainfall areas (typically 350–550 mm/yr., or 12–18 in./yr.). The WWF system has winter wheat planted in the fall and harvested in mid-summer of the following year, with no crop planted the next season, to restore soil moisture. Thus, a crop is typically planted and harvested on only half of the available land each year. The alternative system we analyze here, denoted WWC, involves replacing the fallowed land with *Camelina* so that half of the land is planted to winter wheat each year and half is planted to *Camelina* in a rotation. Experimental data from the region show that this rotation would likely result in a reduction in winter wheat yields from the regional average of about 50 bu/ac to about 33 bu/ac on average, with an average *Camelina* yield of 1400 lb per acre (Wysocki, 2015).

The next step for the TOA-MD analysis is to construct similar data for the alternative WWC system. As we noted in the previous section, if *AgBiz Logic* were used to generate data for this alternative system, participating farmers would be provided available information about *Camelina* such as experimental yields, and the farmers would provide estimates of the yields they would expect to obtain, along with estimates of costs of production for the practices they would implement. To represent the data that could be obtained from *AgBiz Logic*, we use data obtained from enterprise budgets constructed by farmers and extension economists (Seavert et al., 2012, Stein, 2012), and experimental yield data for *Camelina* cited above. Based on experimental data showing that elimination of fallow would reduce wheat yields 35%, the revenue per acre for winter wheat is decreased accordingly, and the cost of production is reduced because fallow costs are eliminated. In place of the fallow, the *Camelina* crop is assumed to yield 1400 lb per acre, and there are similar cost components as noted above for winter wheat (variable costs and machinery replacement). The result is a net return that varies with the price assumed for *Camelina* as shown in Table 2. A low *Camelina* price of $0.10/lb would provide a net return to the WWC system similar to the WWF system. Recent market prices for oilseeds similar to *Camelina* have been in the range of $0.15/lb.

**Table 2:** Mean net returns, adoption rates and treatment effects based on TOA-MD analysis of the Winter wheat – *Camelina* system in the U.S. Pacific Northwest.

*Camelina* price	Mean net returns				
	WWF	WWC	Adoption Rate	ATE	ATT
			Large Farms		
0.1	275	152	21	-45	31
0.15	275	285	52	4	49
0.225	275	485	85	76	97
0.3	275	684	94	148	161
			Small Farms		
0.1	32	17	33	-46	73
0.15	32	41	59	29	115
0.225	32	78	79	145	209
0.3	32	114	86	260	321

Note: WWF = winter wheat-fallow system, WWC = winter wheat-*Camelina* system. Mean net returns are $1000/farm. ATE = average treatment effect = percent change in mean returns between system. ATT = average treatment effect on the treated (adopters) = percent difference between mean return to adopters of WWC and the counterfactual return adopters would receive from WWF. ATE and ATT are in percent of mean returns to WWF.

For analysis of the adoption of a new system using the TOA-MD model, we need estimates of average returns which we interpret as the data from the enterprise budgets described above, and we also need an estimate of the variance of economic returns in the farm population. The use of *AgBiz Logic* to collect data for this scenario from farmers would provide an estimate of this variance. Lacking these data, we assume that the coefficient of variation of *Camelina* returns in the population is similar to the coefficient of variation of returns to winter wheat, and combine that estimate with the estimate of average returns to calculate a variance. The TOA-MD model also requires a value for the correlation between the returns to the WWC and WWF systems. This parameter also could be estimated from data generated by farmers using *AgBiz Logic* to evaluate the WWC system. Lacking these data, we set the value to 0.75, a typical value for this parameter when it can be estimated with observational data.

Table 2 summarizes the average net returns for the WWF and WWC systems, for small and large farm groups, predicted adoption rates and economic impact obtained from the TOA-MD model for *Camelina* prices ranging from a low value of $0.10 to a high value of $0.30 per pound. Prices in the range of $0.10 to $0.15 per pound result in relatively low adoption rates of 20 to 60%, whereas at prices above $0.20/lb adoption would increase to 80–95%, depending on farm size. Thus, the analysis shows that adoption of the WWC system would increase substantially if prices were in this higher range. It is difficult to know what the market price of *Camelina* would be if a biofuel market were developed, but this analysis shows that a price substantially above the recent oilseed market price would be required to induce a high rate of adoption of the WWC system. The analysis also shows somewhat higher adoption rates for larger farms. Examination of the WWF data shows larger farms earn a larger proportion of their income from wheat, and thus benefit relatively more when *Camelina* becomes profitable at high prices, compared to smaller farms that earn somewhat more of their income from non-wheat crops and government subsidies.

The economic impacts of the WWC system are represented in two ways in Table 2. The middle column of the table presents the “average treatment effect” (ATE) which is the average impact of the WWC system relative to the WWF system if it were adopted by all farms (or by a random sample of farms). However, adoption is not random for farmers who choose the system with the highest expected economic returns. Under this behavior, the economic impact on the adopters is measured by the average treatment effect on the treated (ATT), the last column of Table 2. The ATT is equal to the difference between the returns from the WWC system for the adopters and the returns the adopters would receive from the WWF system if they did not adopt.

To summarize, using the results in Table 2, two important implications for the economic impacts of the WWC system are: (1) the average return of this system (the average treatment effect, or ATE) is negative for low *Camelina* prices, meaning that the WWF system would provide higher returns than the WWC on average in the farm population. It follows that the adoption rate will be less than 50% as Table 2 shows for the relatively low *Camelina* price of $0.10; and (2) for those who do adopt WWC, the return is necessarily positive and increases with the *Camelina* price, as indicated by the average treatment effect on the treated, or ATT.

**Fig. 4:**
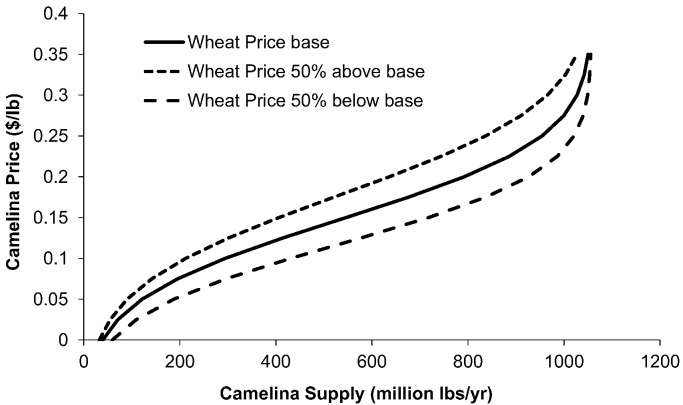
*Camelina* supply curves for the winter-wheat-fallow region of the U.S. Pacific Northwest Based on TOA-MD Model simulations for adoption of the Winter-wheat-*Camelina* system.

Fig. 4 presents the implications of the TOA-MD analysis for *Camelina* supply. By running the simulations for a range of prices, we estimate the willingness of farmers to switch from the WWF to the WWC systems and thus increase *Camelina* production by replacing fallow acres with *Camelina*. The figure shows a relatively elastic response to the *Camelina* price in the $0.10 to $0.225 range. The figure also shows how the supply would be affected by lower or higher wheat prices, with higher wheat prices discouraging WWC adoption. Under our assumption that wheat yields would be reduced, WWC adoption would also result in lower wheat production, reducing it by up to 18% at a high *Camelina* price and a low wheat price. This finding shows that there would be a tradeoff between biofuel production and food grain production. This tradeoff is of interest to policy decision makers, such as the United States Navy, who are concerned about the effect that biofuels could have on food prices. Coupling this analysis to a market equilibrium analysis would provide further information about the possible price and economic impacts of a policy supporting biofuels, e.g., as in Reimer and Zheng (2015).

## 6 Concluding observations

In this paper we describe two analytical tools – *AgBiz Logic* and TOA-MD – that demonstrate the current capability of farm-level and landscape-scale models to meet the needs for improved data, models and knowledge products. In their present form, these models provide substantial capability to address the data challenges identified by Jones et al. (2017), Antle et al. (2017a), and Janssen et al. (2017). However, there are also needs for these and similar models to be improved.

First, models need to be more useful to farm decision makers. As Antle et al. (2017a) observe, users do not want models *per se*, rather they want the information they can produce. This means that models must be embedded in decision support tools that have value to farm managers. One improvement could be to automate data collection using sensors on machinery and other mobile devices, as well as from web-based sources such as weather, and economic data such as prices. Another area of improvement is inter-operability of tools like *AgBiz Logic* with farm accounting and tax preparation software, so that information can be entered once and then utilized in an integrated way across multiple analytical tools. Another area for improvement is inter-operability with other models or model output databases, such as crop simulation models and environmental impact models.

Similar ease-of-use and inter-operability issues apply to analytical tools for landscape-scale analysis like TOA-MD and other simulation models, such as crop or environmental process models that may be used with it. Data from tools like *AgBiz Logic* needs to be integrated with cloud-based systems and with the other public data needed to implement a landscape scale analysis identified in Fig. 3. The current approach of manually carrying out this integration on a case-by-case basis makes this type of analysis costly even in a small geographic region, and often makes integration infeasible across larger regions.

Second, the fact that virtually all stakeholders want access to model outputs, rather than access to the models themselves, means that there is a demand for “knowledge products,” i.e., tools that facilitate access to model outputs and provide analytical capability to interpret model outputs for decision making. As Janssen et al. (2017) observe, it remains to be seen what form these knowledge products will take – as “apps” on mobile devices, as is now being done for some types of decision making such as pesticide spray decisions – or as larger tablet computer dashboards for data visualization and additional processing through meta-models and other analytical tools. The fact that these knowledge products have been slow to materialize suggests some form of “market failure” – i.e., some constraints that prevent this latent demand from being expressed and satisfied.

A perusal of the rapidly emerging market for private advisory services utilizing “big data” shows that, at least in some parts of the world such as the United States where large-scale commercial agriculture dominates, this latent demand is beginning to be met by private industry (Antle et al., 2015a, Antle et al., 2015b). Yet it remains to be seen how this emerging private supply of information services will operate, and whether it can also satisfy the public good uses of such data. It is even less clear how these technologies can serve the needs of the small-scale farmers in the developing world where commercialization lags.

As suggested by Antle et al. (2017a), one solution to these challenges appears to be private-public partnerships among the various organizations that have a mutual interest in assuring that the data are obtained efficiently and used appropriately for both private and public purposes. Such partnerships could help create a pre-competitive space for the development of data and analytical tools that is built on the recognition that there are important public-good attributes of the data, methods and analytical tools, linked to a competitive space to incentivize the commercial development of improved knowledge products.

Several challenges need to be addressed to facilitate a linkage between farm-level management tools such as *AgBiz Logic* and policy analysis tools like TOA-MD and other landscape scale models. First, a statistically representative group of farms would need to be identified who would agree to use *AgBiz Logic* and allow their data to be used in a landscape scale analysis. This would involve a sampling process similar to identifying a sample of farms for a farm-level economic survey. Second, software would need to be designed to transmit and assemble the individual farm data into a database that could subsequently be used to estimate TOA-MD parameters while maintaining confidentiality of individual producers. Note that data would need to be collected over multiple growing seasons in most cases to account for crop rotations and other dynamic aspects of the farming system. Farm household characteristic data could be collected as a part of *AgBiz Logic*, or could be collected using a separate survey instrument. Environmental and social outcome data collection would need to be tailored to the specific type of variable. For example, measurement of soil organic matter could require infield soil sampling and laboratory analysis, possibly combined with modeling, or the use of specialized sensors.

For scenario analysis, it is necessary to project from current biophysical and socioeconomic conditions into the alternative conditions described by a scenario. For climate impact assessment, this is currently being done on a global scale using new scenario concepts called “Representative Concentration Pathways” and “Shared Socio-Economic Pathways.” To translate these future pathways into ones with more detail needed for agricultural assessments, “Representative Agricultural Pathways” are being developed (Valdivia et al., 2015). The data acquired through tools such as *AgBiz Logic* could be combined with these future projections to implement regional integrated assessments using the methods developed by the Agricultural Model Inter-comparison and Improvement Project (Antle et al., 2015a).
